# When does mutualism offer a competitive advantage? A game-theoretic analysis of host–host competition in mutualism

**DOI:** 10.1093/aobpla/plac010

**Published:** 2022-03-10

**Authors:** Abdel H Halloway, Katy D Heath, Gordon G McNickle

**Affiliations:** 1 Department of Plant Biology, University of Illinois at Urbana-Champaign, 505 S. Goodwin Avenue (M/C 116), Urbana, IL 61801, USA; 2 Department of Botany and Plant Pathology, Purdue University, 915 W. State Street, West Lafayette, IN 47907, USA; 3 Carl R. Woese Institute for Genomic Biology, University of Illinois, 1206 W. Gregory Drive, Urbana, IL 61801, USA; 4 Purdue Center for Plant Biology, Purdue University, 915 W. State Street, West Lafayette, IN 47907, USA

**Keywords:** Evolutionary game theory, evolutionarily stable strategies, matrix game, mutualism, mycorrhizae, plant–microbe interactions

## Abstract

Due to their non-motile nature, plants rely heavily on mutualistic interactions to obtain resources and carry out services. One key mutualism is the plant–microbial mutualism in which a plant trades away carbon to a microbial partner for nutrients like nitrogen and phosphorous. Plants show much variation in the use of this partnership from the individual level to entire lineages depending upon ecological, evolutionary and environmental context. We sought to determine how this context dependency could result in the promotion, exclusion or coexistence of the microbial mutualism by asking if and when the partnership provided a competitive advantage to the plant. To that end, we created a 2 × 2 evolutionary game in which plants could either be a mutualist and pair with a microbe or be a non-mutualist and forgo the partnership. Our model includes both frequency dependence and density dependence, which gives us the eco-evolutionary dynamics of mutualism evolution. As in all models, mutualism only evolved if it could offer a competitive advantage and its net benefit was positive. However, surprisingly the model reveals the possibility of coexistence between mutualist and non-mutualist genotypes due to competition between mutualists over the microbially obtained nutrient. Specifically, frequency dependence of host strategies can make the microbial symbiont less beneficial if the microbially derived resources are shared, a phenomenon that increasingly reduces the frequency of mutualism as the density of competitors increases. In essence, ecological competition can act as a hindrance to mutualism evolution. We go on to discuss basic experiments that can be done to test and falsify our hypotheses.

## Teaser

Plants frequently partner with microbes to obtain essential nutrients. Plants show variation in this mutualistic partnership, particularly with ecological and environmental context. We created a model of individual plant fitness given an ecological background to see how competition between plants over microbially derived resources affects this mutualism’s evolution. Our results show that such competition can hinder the evolution which may be relevant for some partnerships (e.g. mycorrhizal fungi) but not others (e.g. rhizobia). Our model also points to simple experiments which could be used to falsify our hypothesis.

## Introduction

The non-motile nature of plants means they frequently rely on other organisms to carry out functions such as seed dispersal, pollination and nutrient acquisition (Howe and Westley 1990). Two key nutrient acquisition strategies for plants are the microbial symbioses with mycorrhizae (in 80 % of plant species and 92 % of plant families; Simon *et al.* 1993; Wang and Qiu 2006) and symbiotic nitrogen-fixing bacteria (in a smaller subset of families; de Faria *et al.* 1989; Sprent 2005). In these mutualisms, the plants trade carbon in the form of carbohydrates and lipids while receiving nutrients like nitrogen and phosphorous (Hawkins *et al.* 2000; Hodge *et al.* 2001; Sessitsch *et al.* 2002; Sawada *et al.* 2003; Leigh *et al.* 2009). Across the plant kingdom, the commonality of partnering with microbial mutualists implies that doing so often offers a fitness benefit to plants (Hartnett *et al.* 1993). However, it is also known that the costs and benefits of mutualism depend upon ecological and evolutionary factors such as nutrient availability and frequency of plant genotypes (Peng *et al.* 1993; Heath and Tiffin 2007; Bronstein 2009; Chamberlain *et al.* 2014; Lu and Hedin 2019). These variations in benefits can have knock-on effects at larger scales leading to the variation in the presence or absence of the mutualist partnership among lineages (de Faria *et al.* 1989; Werner *et al.* 2015; Maherali *et al.* 2016). In this paper, we sought to determine how ecological and environmental context, particularly competitive interactions and nutrient availability, respectively, could promote or exclude the mutualistic partnership and ultimately lead to its evolution in a species, and whether populations might ever stably contain a mixture of mutualist and non-mutualist individuals.

To understand how context determines evolution of microbial mutualisms, we turned to mathematical analysis. Mathematical analysis has been widely used to understand the evolution and persistence of mutualism (Noë and Hammerstein 1995; Ferriere *et al.* 2002; West *et al.* 2002; Hoeksema and Kummel 2003; Akçay and Roughgarden 2007; Akçay and Simms 2011). Typically, the focus of these models has been on the stability and maintenance of interactions between partners, the host and the symbiont, with reasons such as partner selection (West *et al.* 2002; Akçay and Roughgarden 2007; Akçay and Simms 2011) and spatial structure given (see Wilson *et al.* 2003 for a model of seed dispersal). While these models may implicitly assume competition between host plants, they do not explicitly model the nature of the competition between plants that might vary with mutualism strategy, or ecological and density effects (Friesen and Jones 2012). We sought to understand how the nature of this host–host competition may determine the evolution of mutualism, especially in the context of mutualism offering a competitive advantage to a host (Jones *et al.* 2012). To do so, we turned to evolutionary game theory. Originally developed to understand animal behaviour, evolutionary game theory is a mathematical framework that examines how strategies perform, in terms of fitness, against other interacting strategies (Maynard-Smith and Price 1973; Geritz *et al.* 1998; Brown 2016). It has been applied widely across taxa; for plants, it has been used to understand properties such as defence against herbivory and biomass allocation with competition (Givnish 1982, 1995; Augner *et al.* 1991; McNickle *et al.* 2016). Recently, evolutionary game-theoretic host–host competition has been used to understand the global distribution of nutrient acquisition strategies (Lu and Hedin 2019). Viewing the partnership with microbes (and its complement, non-partnership) as strategies in an evolutionary game narrows our focus to just the competitive interactions between hosts how they depend on biotic and abiotic factors.

To this end, we created a 2 × 2 matrix game to determine how nutrient availability, frequency of alternate strategies and competitor density may (or may not) offer an intraspecific competitive advantage to a plant that partners with a microbe to obtain nutrients. In our model, we assume that the mutualism partnership is itself a strategy, the equivalent of a functional trait (Violle *et al.* 2007), where a plant can either be a non-mutualist and only acquire benefits from freely available nutrients in the soil or be a mutualist and receive additional benefits from microbially obtained nutrients. All plants must pay a cost to acquire the freely available nutrients with mutualists paying an additional cost for the microbially obtained nutrients. Besides these four parameters, we also included local competitor number as a parameter to see how density-dependence may influence selection (Clarke 1972; Lande *et al.* 2009). We analysed our game for the fixation of either strategy as well as coexistence of both strategies within a population. We discuss what our results mean for the evolution of and variation in mutualist strategies in plant–microbe systems.

## Model Analysis

### Competition with one plant

In our model, we start out by assuming there are two pools of nutrients available to a plant: one that is freely available *AN* and one that is only obtained through microbial mutualism *MN*. These nutrients provide fitness benefits of *B*_*AN*_ and *B*_*MN*_, respectively, to a plant. Some proportion of the population is the genotype of plants with the ability to partner with microbial mutualists while the remainder is made up of the genotype that cannot; we hereafter refer to those genotypes as mutualists and non-mutualists, respectively. Non-mutualist plants only get the fitness benefit from the freely available nutrients while mutualists get fitness benefits from both freely available nutrients and microbially obtained nutrients. All plants must produce roots to obtain the freely available nutrient at a cost of *c*_*r*_. Mutualists, however, have to pay an additional fitness cost *c*_*t*_ to obtain the microbial nutrients due to trade and other mechanisms (e.g. allocation of biomass to nodules in the case of rhizobia mutualism). Finally, we begin our analysis by assuming only two plants compete at a given instant with each plant having equal competitive ability. From these assumptions, we construct the following fitness matrix for each type of plant:

**Table:** 

		Resident	
		Non-mutualist	Mutualist
Focal invader	Non-mutualist	$\frac{B_{AN}}{2}-c_{r}$	$\frac{B_{AN}}{2}-c_{r}$
	Mutualist	$\frac{B_{AN}}{2}-c_{r}+B_{MN}-c_{t}$	$\frac{B_{AN}}{2}-c_{r}+\frac{B_{MN}}{2}-c_{t}$

Since all individuals regardless of strategy have access to freely available nutrients, they will compete over that pool of nutrients. Assuming that they are equally strong in competitive ability, all individuals receive exactly half of the potential fitness benefits from this pool of resources $\frac{B_{AN}}{2}$. We also assume that all individuals must produce the same amount of roots for the same cost *c*_*r*_; therefore, all individuals, mutualist and non-mutualist, have a base net fitness benefit of $\frac{B_{AN}}{2}-c_{r}$. Mutualists, however, have access to microbially obtained nutrients and therefore receive a benefit from those nutrients while paying the cost in the form of resources traded away. If a mutualist is with a non-mutualist, the mutualist gets the full benefit of the microbially obtained nutrients while paying the cost of trade $B_{MN}-c_{t}$; however, when with another mutualist, both compete over and therefore equally share the microbially obtained nutrients leading to a net benefit of $\frac{B_{MN}}{2}-c_{t}$.

Since all individuals receive the exact same fitness benefit from the freely available nutrient and pay the exact same cost for the roots $\frac{B_{AN}}{2}-c_{r}$, these terms can be removed to arrive at the simpler pay-off matrix below:

**Table:** 

		Resident	
		Non-mutualist	Mutualist
Focal invader	Non-mutualist	0	0
	Mutualist	$B_{MN}-c_{t}$	$\frac{B_{MN}}{2}-c_{t}$

**Figure 1.**
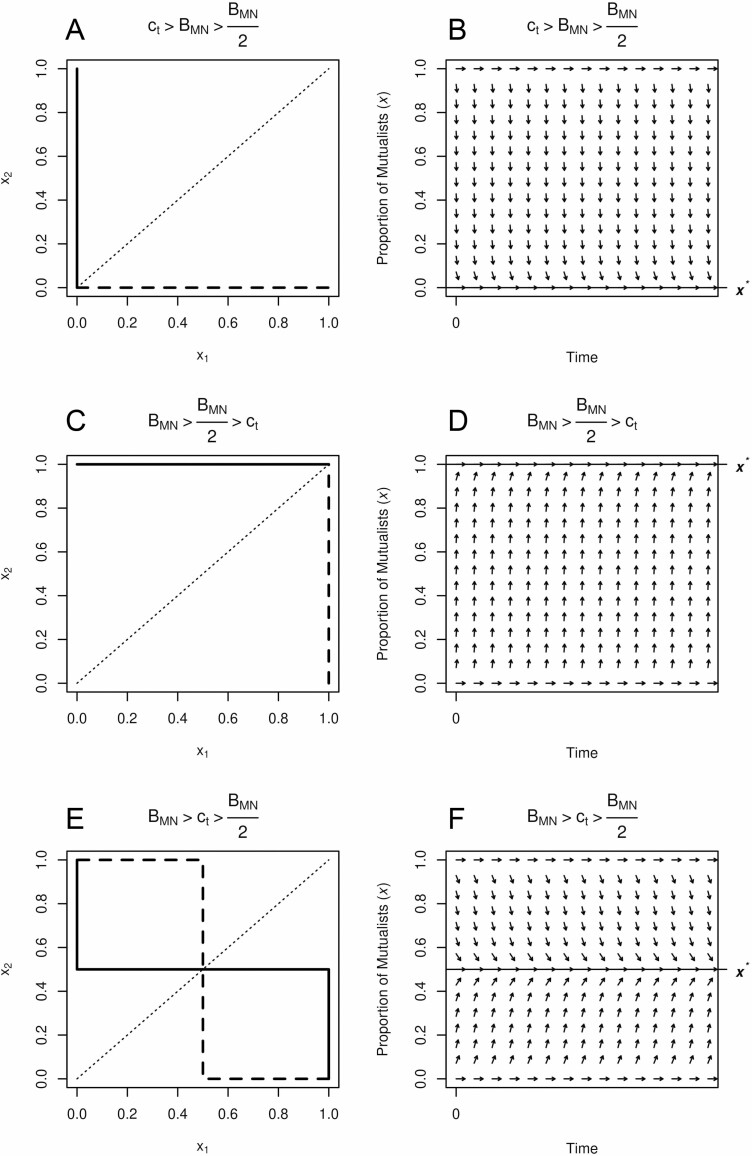
Evolutionary dynamics as seen through best response curves (A, C, E) and directional fields (B, D, F) for the three qualitatively different scenarios. In the first scenario (A, B), the cost of mutualism outweighs any benefit regardless of the opposing player’s strategy. In the second scenario (C, D), the benefit of mutualism outweighs the cost regardless of the opposing player’s strategy. In the third scenario (E, F), the benefit of mutualism outweighs the cost only when the opposing player is a non-mutualist. Results are shown specifically for $x^{*}=0.5$ ($c_{t}=1$ and $B_{MN}=4$) but generally apply to $0>x^{*}>1$. For the best response curves (A, C, E), $x_{i}$ indicates the best strategy for the *i*th player with greater values of $x_{i}$ indicating mutualism. Solid lines are the best response for player 1 and dashed lines for player 2. As this is an intraspecific evolutionary game of a single population, the dotted line $x_{1}=x_{2}$ indicates the feasible set of solutions. Actual solutions for $x^{*}$ are the intersection of all three lines. (A) The best response leads to a single strategy evolutionarily stable strategy (ESS) of non-mutualism fixation. (C) The best response leads to a single strategy ESS of mutualism fixation. (E) The best response leads to a multiple strategy ESS of coexistence between mutualism and non-mutualist types. Replicator dynamics show the same results as the best response curves (B, D, F); the only difference is that fixation of either strategy is an equilibrium in all three scenarios but the stability of those two equilibria varies according to the cost–benefit ratio.

**Figure 2.**
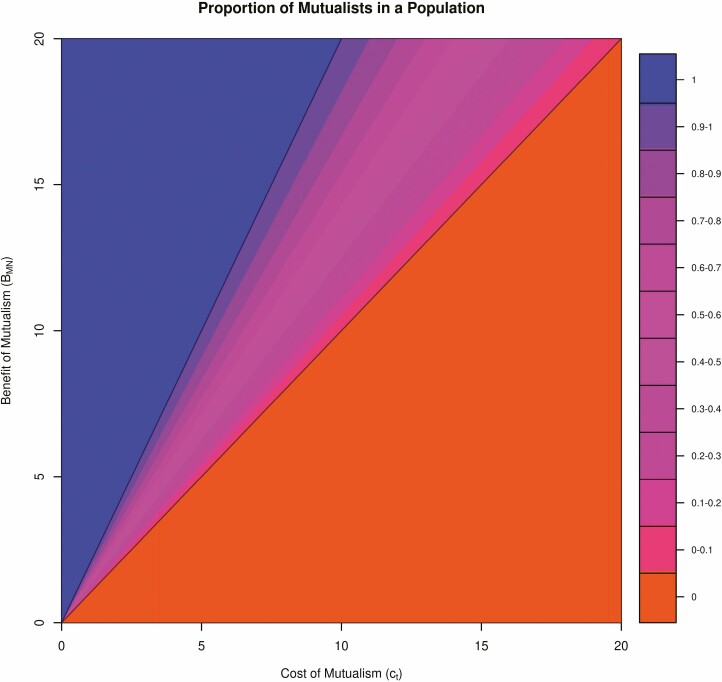
A plot of the proportion of mutualists in a population $x^{*}$ for combinations of $B_{MN}$ and $c_{t}$. The orange-red region below and to the right of both lines indicates non-mutualist fixation, the blue region above and to the left of both lines indicates mutualist fixation and the magenta region between both lines indicates coexistence.

From this simplified matrix, we can quickly arrive at conditions for fixation of mutualist or non-mutualist varieties. Specifically, if the cost of trade outweighs the total benefit of microbially obtained nutrients $c_{t}>B_{MN}$, then mutualists do worse, and non-mutualism is the dominant strategy (Fig. 1A and B). This is intuitive and true of any trait: when the fitness costs outweigh the benefits, no trait should be favoured by natural selection. However, if the benefits of microbially obtained nutrients after competition with other mutualist plants in the population are greater than the cost of trade $\frac{B_{MN}}{2}>c_{t}$, then mutualists always do better and so become the dominant strategy (Fig. 1C and D). Interestingly, even in this most basic model the difference between $B_{MN}$ and $\frac{B_{MN}}{2}$ creates a region of the fitness landscape where mutualists and non-mutualists can coexist within the same population. Indeed, if the total benefit of microbially obtained nutrients is greater than the cost of trade but the benefit of microbially obtained nutrients under competition is lower than the cost of trade (i.e. $B_{MN}>c_{t}>\frac{B_{MN}}{2}$), then both genotypes coexist in the same shared space (Fig. 1E and F). Solving for the equilibrium proportion of mutualists in the population gives $x^{*}=2\left(1-\frac{c_{t}}{B_{MN}}\right)$ (Fig. 2). This coexistence point is an stable equilibrium making it an evolutionarily stable strategy (ESS), defined as the composition of strategies in a population which cannot be invaded by a new, different strategy (Fig. 1F).

### Competition and neighbourhood size

Above, we assumed that plants competed with only one other individual at a given time. While the non-motile nature of plants means that they compete on local spatial scales, this neighbourhood of competitive interactions is generally more than one neighbour. It can be especially true when nutrients are scarce and multiple individuals must draw from the same pool leading to each individual taking up a smaller share of nutrients. For a mutualist plant, its share of the microbially available nutrients will also depend on the frequency of mutualists in the neighbourhood which ultimately depends on the frequency of mutualists in the population. Therefore, we modify our game to have a plant compete with any number of individual plants in its local neighbourhood. We can generalize our fitness matrix such that

**Table:** 

		Resident neighbourhood (*n*)		
		Purely non-mutualist	Mixed neighbourhood	Purely mutualist
Focal invader	Non-mutualist	$\frac{B_{AN}}{n+1}-c_{r}$	$\frac{B_{AN}}{n+1}-c_{r}$	$\frac{B_{AN}}{n+1}-c_{r}$
	Mutualist	$\frac{B_{AN}}{n+1}-c_{r}+B_{MN}-c_{t}$	$\frac{B_{AN}}{n+1}-c_{r}+\frac{B_{MN}}{xn+1}-c_{t}$	$\frac{B_{AN}}{n+1}-c_{r}+\frac{B_{MN}}{n+1}-c_{t}$

where *n* is the number of competitors per plant, i.e. the size of its local neighbourhood, and *x* is the frequency of mutualists in that neighbourhood. Like before, fitness benefits from freely available nutrients are invariant with strategy. Therefore, it can be subtracted from each expression to arrive at the simpler matrix below.

**Table:** 

		Resident neighbourhood (*n*)		
		Purely non-mutualist	Mixed neighbourhood	Purely mutualist
Focal invader	Non-mutualist	0	0	0
	Mutualist	$B_{MN}-c_{t}$	$\frac{B_{MN}}{xn+1}-c_{t}$	$\frac{B_{MN}}{n+1}-c_{t}$

**Figure 3.**
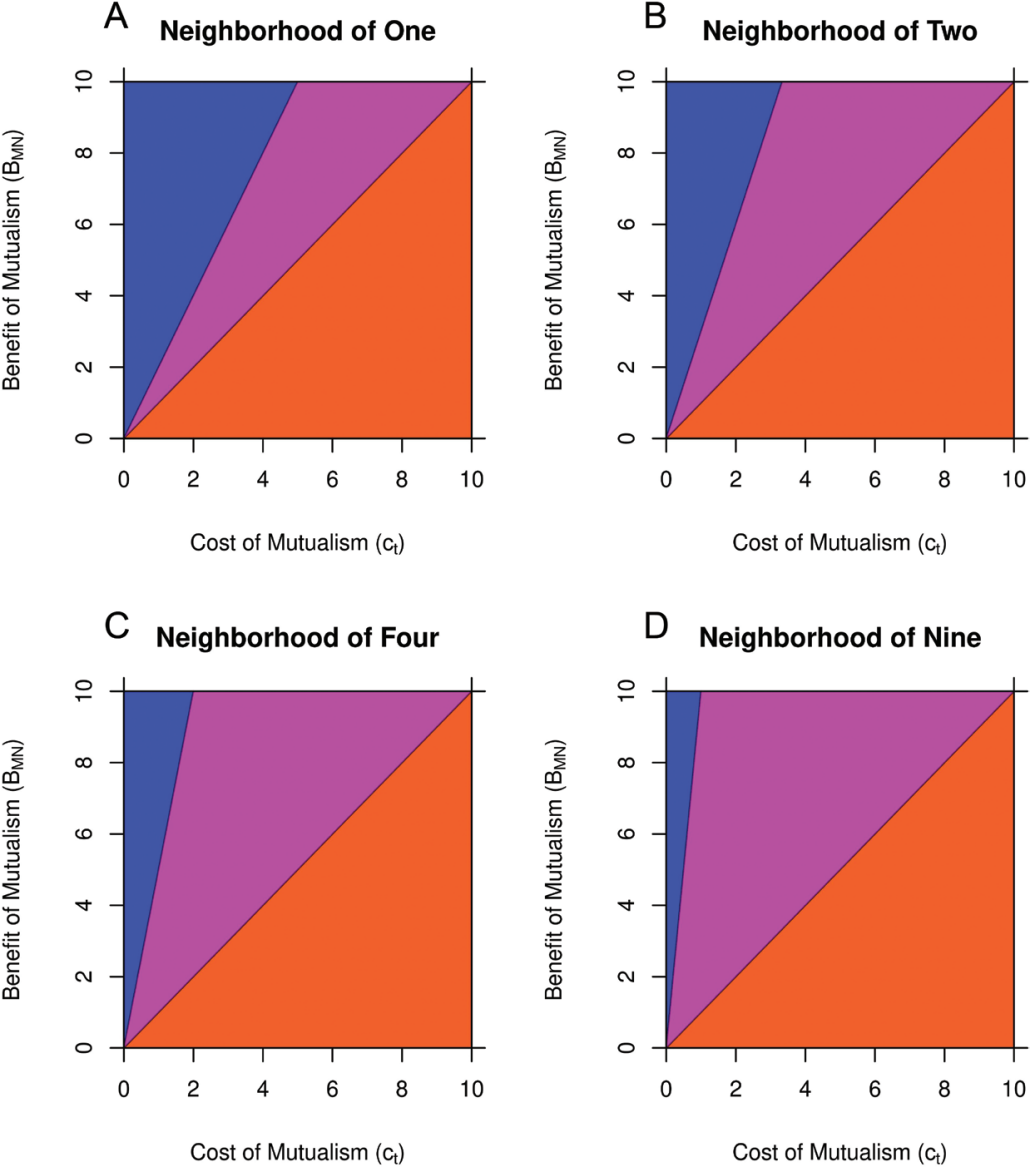
Plots of how regions of coexistence change with increasing neighborhood size from part A to part D. The colours remain the same as Fig. 2. The region of fixation for the non-mutualist strategy does not change with neighbourhood size and the same is true for the region where mutualist strategy is present, i.e. the combined region of mutualist fixation and coexistence. However, the region of mutualist fixation becomes smaller, expanding the region of coexistence between strategies.

Following from Hauert *et al.* (2006), we derive overall fitness of a mutualist plant to be $\frac{B_{MN}(1-{\left(1-x\right)}^{n+1})}{x(n+1)}-c_{t}$ assuming local neighbourhoods are generated randomly (**see****Supporting Information** for derivation). With a larger neighbourhood of interaction, the criterion for non-mutualist fixation is unchanged and still requires that the cost of mutualism without mutualist competitors must be greater than the benefits $c_{t}>B_{MN}$. Fixation of the mutualist strategy requires that the benefit of mutualism when solely competing with mutualist must be greater than the costs $\frac{B_{MN}}{1+n}>c_{t}$. We can express this criterion in terms of a cost–benefit ratio $\frac{B_{MN}}{c_{t}}>n+1$. From this ratio, we can see that as *n* increases, there needs to be a corresponding increase in benefits relative to the costs, reducing the possibility of fixation of the mutualist strategy within a population. This means that mutualist strategy is more likely to appear in coexistence with the non-mutualist strategy with an increasing number of competitors (Fig. 3). Solving for this coexistence equilibrium proportion of mutualists is significantly harder with multiple competitors, and is analytically impossible with five or more individuals, but we can arrive at the solution $x^{*}=\frac{1}{2}\left(3-\sqrt{12\frac{c_{t}}{B_{MN}}-3}\right)$ when there is a neighbourhood of two plant competitors (**see****Supporting Information** for the solution for three competitors).

## Discussion

In this study, we explored how competitive interactions and nutrient availability could lead to an intraspecific competitive advantage for a plant that partners with a microbe. Many models of mutualism evolution focus on the stability of the plant–microbe partnership, especially with regard to microbial cheating and the maintenance of beneficial variants (West *et al.* 2002; Akçay and Roughgarden 2007; Akçay and Simms 2011). Host–host interactions are usually not a focus in these models of evolution but rather are treated implicitly (Bergstrom and Lachmann 2003) (however see Lu and Hedin 2019). Our model explicitly focused on host–host competition and the competitive advantage for a host plant. As a basic check against previous work, our model also shows the intuitive result that if the cost of mutualism outweighed the benefit, then non-mutualists would entirely exclude mutualist. Alternatively, if the benefit of mutualism was greater than the cost under at least some conditions, then mutualism would be a viable strategy. That evolution favours traits with higher benefits compared to costs is well known, but by including density dependence to create eco-evolutionary feedback loops, we gained more precise insight into how benefits and costs combined within the context of intraspecific plant competition shape the evolution of mutualism. In particular, we show how frequency dependence can lead to reduced benefits of mutualism when mutualist resources are shared, a result that is strengthened by our analysis of density effects and competitor number (Fig. 3). Quite simply, ecological competition in our model acts as a hindrance to mutualism evolution. Thus, given that plants can compete with a significant number of neighbours especially when the resource is motile like nitrogen, phosphorous or potassium (Silander and Pacala 1985; Goldberg 1987; Casper and Jackson 1997; Stoll and Weiner 2000), our model predicts that mutualist and non-mutualists should frequently coexist within the same population. This hypothesis that there might frequently be mixed strategies of mutualist and non-mutualist genotypes, or of genotypes of varying levels of investment in mutualism and number of mutualist partners, stably coexisting within the same local population is one that is not generally tested even though it is a robust prediction from our model.

Our model was simple. It assumed that the benefits and costs of obtaining nutrients were constant, only changing with competition between host plants. Because all host plants competed equally for the same freely available nutrients regardless of strategy, it had no effect on our results. All that mattered was the net benefit of mutualism, which mostly depends on the fitness gains from microbially obtained nutrients and fitness losses from microbial trade. This conflicts with at least some empirical studies showing that increasing nitrogen availability leads to a reduction in mutualism, both ecologically in a community or evolutionarily in a population (Vitousek *et al.* 1997; Weese *et al.* 2015; Regus *et al.* 2017; McCoy *et al.* 2018; Taylor and Menge 2018) (but see Simonsen *et al.* 2015; Wendlandt *et al.* 2022). Thus, most evidence to date suggests that microbial mutualism does not simply occur as an added benefit to the plant, but instead implies some trade-off between using freely available nutrients and microbially obtained nutrients. This could be due to a fixed resource budget on the part of the plant—anywhere between 4 and 20 % of total plant carbon is traded to mycorrhizal partners (Johnson *et al.* 1997; Voisin *et al.* 2003; Taylor and Menge 2018)—varying marginal costs of investment in the sources of the nutrients, preference for the form of the nutrient (Falkengren-Grerup 1995) or some combination of the three. Trade-offs would also affect how competition over the freely available nutrients occurs with mutualists being worse competitors for those nutrients. This would make mutualism harder to evolve but may paradoxically make coexistence even more likely with neighbourhood size as non-mutualist fixation becomes harder with increasing neighbourhood size.

The most interesting result of our model is that coexistence only happened if mutualists competed for the same microbially obtained nutrients. If the plants did not compete for the same mutualism-derived nutrients, then there would only be fixation of either strategy as either could be competitively dominant. We know that some microbial mutualisms differ in their nutrient sources. Mycorrhizae obtain their traded nutrients such as phosphorous and nitrogen from organic sources (Hawkins *et al.* 2000; Hodge *et al.* 2001; Leigh *et al.* 2009), a depletable resource likely shared between mutualist competitors. Rhizobia, on the other hand, get their traded nitrogen from fixing atmospheric nitrogen, a functionally unlimited resource that likely is not locally depletable (Sessitsch *et al.* 2002; Sawada *et al.* 2003). In the rhizobial mutualism, benefits may not change in the presence of competitors with the same strategy. This lack of sharing the microbially derived resources may add to the explanation as to why legumes are so dominant in mutualistic invasions compared to mycorrhizal-associated plants (Richardson *et al.* 2000; Castro-Díez *et al.* 2014). If a mutualist invader must share its resources with other competitors, it becomes limited by its own success; with more individuals using the same strategy, frequency dependence puts an upper limit on how successful an invader can be, especially with a larger neighbourhood of competition. By not having to share resources, invading legumes may represent a purely dominant strategy, at least in the right conditions (i.e. until other resources, such as light availability, become limiting).

Modifications to this model can be made to reveal other aspects of mutualism evolution. For example, we assumed that a plant either was a mutualist and so fully invested in mutualism or was not a mutualist, regardless of whether net benefits were positive or negative. This is likely true at larger scales and interactions at the intertaxonomic level where entire lineages show the presence or absence of mutualism strategies (Sprent 2005; Werner *et al.* 2015). However, at smaller scales of the individual and population, variation in mutualism is likely to present itself in a more continuous and quantitative fashion (Heath and Stinchcombe 2014). The abstract nature of mathematical modelling does mean that our equilibrium proportion $x^{*}$ could be understood as the proportion of mutualists in a population or community depending on whether the interactions are thought to be intra- or interspecific, respectively, as well as probability of any individual using the mutualism strategy or the level of investment in the mutualism. However, different processes and properties operate on these different scales (Jablonski 2008). At the individual level, timescales are within a lifetime, and responses to changing conditions are governed by anatomical and physiological plasticity and variation within that organism. At the population and community level, timescales operate over generations and responses are governed by variation between individuals leading to variation in fitness and reproduction. Both scales are unique but influence each other; seeing how plasticity at the individual level drives variation at the population/community level and vice versa would certainly reveal much about the dynamics of mutualism evolution. Such a model of plasticity in the amount of trade would require more than just fitness benefits of nutrients, it would require a second resource (i.e. carbon) for the plant to trade. We suggest that this model could become a more process-based model of plant growth that includes photosynthesis to acquire carbon for trade as well as nutrient dynamics in soil. A number of models of plant growth with limitation from multiple essential resources exist (Pacala and Tilman 1994; Craine *et al.* 2005; Dybzinski *et al.* 2011; McNickle *et al.* 2016). Future work could explore introducing some of the insights gained in our simple model into those more complex models of plant growth and allocation.

This simplicity of our model does offer an advantage in that it can be easily translated to an experimental set-up for falsification. One potential set-up could be pot experiments with mutualist and non-mutualist varieties of plants (McNickle *et al.* 2020). Some plant species have loss of function mutants that allow for resource mutualisms to be turned on or off such as *DMI1* in *Medicago* and *sym8* in *Pisum* (Markwei and LaRue 1992; Balaji *et al.* 1994; Guinel and Geil 2002; Ané *et al.* 2004). One could grow the mutants and wild type of the same species together in the same space with different densities and nutrient concentrations to derive relative fitness of each variety. Fitness proxies like seed and flower number, average seed size, plant height and root and shoot biomass could be measured for comparisons between wild type and mutants (subsequent statistical analyses would have to account for intrinsic fitness differences between wild type and mutant genotypes, as wild type typically have greater overall condition and subsequently fitness than mutants). Because these mutants do not express mutualisms with both mycorrhizae and rhizobia, comparisons between different microbial partners can also be made. Based upon our model, we would predict that competition for traded nutrients would influence mutant/wild type relative fitness more for mycorrhizal than rhizobial symbiosis.

## Conclusion

Our model, though simple, reveals that a host plant can gain a competitive advantage from partnering with a microbe, and points to the possibility of coexistence of mutualist strategies in a population, an experimentally testable hypothesis. The results elucidate the basic conditions of positive net benefit and low local competition needed for this competitive advantage and how these results depend how microbially obtained nutrients are shared among competing hosts. We suggest that the competitive advantage had from the mutualistic partnerships makes them prevalent but remain variable as this competitive advantage varies based on biotic and abiotic factors. We suggest that future models incorporate mutualism into process-based models of plant growth.

## Supplementary Material

plac010_suppl_Supplementary_Material

## Data Availability

All data and results were generated through analytical methods. All information regarding full analyses can be found in the **Supporting Information**. Code used to make the figures can be found at the Github Digital Repository: http://dx.doi.org/10.5281/zenodo.6170514.

## Notes

**Evolution & Diversity. Chief Editor**: Jeremy Beaulieu
