# Supplementary material for: When does mutualism offer a competitive advantage? A game-theoretic analysis of host–host competition in mutualism
Source: AoB Plants. 2022 Mar 10;14(2):plac010. doi: 10.1093/aobpla/plac010 (PMC9015964; doi:10.1093/aobpla/plac010)
Supplement: plac010_suppl_Supplementary_Material [file plac010_suppl_supplementary_material.pdf]

Supplementary Information

In this supplementary information, we show the derivation of analytical solutions for our evolutionary game as referenced in the main text.

Competition with one plant

From the main text, the payoff matrix for competition with one plant looks like this:

		Resident	
		Non-Mutualist	Mutualist
Focal Invader	Non-Mutualist	0	0
	Mutualist	$B_{MN} - c_t$	$\frac{B_{MN}}{2} - c_t$

We denote the equilibrium proportion of mutualists as x^*. In this world, the fitness of a mutualist individual is $(1 - x^*)(B_{MN} - c_t) + x^* \left(\frac{B_{MN}}{2} - c_t\right) = \frac{B_{MN}}{2}(2 - x^*) - c_t$. The fitness of a non-mutualist individual is 0. At equilibrium, the fitness of both individuals will be equal, and therefore simplifying the equation $\frac{B_{MN}}{2}(2 - x^*) - c_t = 0$ yields $x^* = 2\left(1 - \frac{c_t}{B_{MN}}\right)$.

Competition and neighborhood size

The payoff matrix for competition with multiple plants from the main text is:

		Resident Neighbourhood (n)		
		Purely Non-Mutualist	Mixed Neighbourhood	Purely Mutualist
Focal Invader	Non-Mutualist	0	0	0
	Mutualist	$B_{MN} - c_t$	$\frac{B_{MN}}{xn + 1} - c_t$	$\frac{B_{MN}}{n + 1} - c_t$

Following Hauert et al. (2006), the fitness of an individual is the probability that it finds itself in a neighborhood with a certain composition multiplied by the fitness of that individual given that neighborhood. Once again, let x^* be the equilibrium proportion of mutualists in a population. The probability that an individual finds itself in a neighborhood of n individuals with i mutualist individuals is $\binom{n}{i}(1 - x^*)^{(n-i)}(x^*)^i$ while its fitness in this neighborhood is $\frac{B_{MN}}{i+1} - c_t$ leading to

a probabilistic fitness of $\binom{n}{i}(1-x^*)^{(n-i)}(x^*)^i\frac{B_{MN}}{i+1}-c_t$. This probabilistic fitness is summed over all probabilities $\sum_{i=0}^{n}\binom{n}{i}(1-x^*)^{(n-i)}(x^*)^i\frac{B_{MN}}{i+1}-c_t$ yielding $\frac{B_{MN}\left(1-(1-x^*)^{n+1}\right)}{x^*(n+1)}-c_t$. The fitness of a non-mutualist individual remains 0.

Because the fitness term is a higher order polynomial, we cannot get an explicit solution for all neighborhood size n. However, smaller neighborhoods can yield explicit solutions. We already derived the solution with a neighborhood of one, $n=1$, above. For a neighborhood of two, the fitness of a mutualist is $\frac{B_{MN}}{3}\left(3-3x+x^{*2}\right)-c_t$. Setting this equation equal to zero (the fitness of the non-mutualist), using the quadratic equation, and selecting only valid solutions give us an equilibrium proportion of mutualists of $x^*=\frac{1}{2}\left(3-\sqrt{12\frac{c_t}{B_{MN}}-3}\right)$. For a neighborhood of three, the fitness of the mutualist is $\frac{B_{MN}}{4}\left(4-6x^*+4x^{*2}-x^{*3}\right)-c_t$ and the equilibrium proportion in the population is $x^*=\frac{1}{3}\left(4+\sqrt[3]{\frac{10B_{MN}-54c_t+6\sqrt{3B_{MN}^2-30B_{MN}c_t+81c_t^2}}{B_{MN}}}-\sqrt[3]{\frac{4B_{MN}}{5B_{MN}-27c_t+3\sqrt{3B_{MN}^2-30B_{MN}c_t+81c_t^2}}}\right)$. A neighborhood of four can also be solved using the same method. However, this derivation is extremely long and not particularly informative. Thus, we do not show it here.

Hauert C, Michor F, Nowak MA, Doebeli M. **2006**. Synergy and discounting of cooperation in social dilemmas. *Journal of Theoretical Biology* **239**: 195–202.
